# Effect of thymoquinone on vitamin D metabolism in glucocorticoid-induced insulin resistance

**DOI:** 10.22038/ijbms.2024.81932.17725

**Published:** 2025

**Authors:** Hazel Berna Göktuğ, Semiha Dede

**Affiliations:** 1 Van Yuzuncu Yil University, Institute of Health Sciences, Van, Turkiye; 2 Van Yuzuncu Yil University, Faculty of Veterinary Medicine, Biochemistry Department, Van, Turkiye

**Keywords:** Glucocorticoid, Insulin resistance, Metabolism, Thymoquinone, Vitamin D

## Abstract

**Objective(s):**

The key ingredient in *Nigella sativa*, thymoquinone (TQ), has several beneficial (antioxidant and anti-inflammatory) properties. This study aimed to investigate the vitamin D metabolism in insulin resistance and the effects of TQ.

**Materials and Methods:**

Male Wistar albino rats were used. TQ was administered as a therapy, and prophylaxis and treatment with metformin were set up for the groups in which insulin resistance had been developed. The gene groups implicated in vitamin D metabolism underwent RT-PCR gene expression analysis and western blot protein analysis.

**Results:**

The analysis shows that the application of TQ reduced HOMA-IR (a sign of insulin resistance). The expression of the VDR gene may be responsible for TQ's effect on treating insulin resistance.

**Conclusion:**

It has been demonstrated that using TQ for therapeutic and preventive reasons is advantageous for improving insulin resistance metrics. Serum vitamin D level was also found to be impacted, which was found to be directly related to the expression of several genes involved in vitamin D metabolism in the liver. However, some of these genes were found to be relatively ineffective in the present study.

## Introduction

In the study evaluating the antidiabetic effects of TQ on streptozotocin (STZ)-induced diabetes in rats, it was determined that TQ significantly improved the integrity of pancreatic islets, glucose-insulin homeostasis parameters, lipid profile markers, and hepato-renal functional and histomorphological conditions, all of which were severely impaired in the untreated diabetic group. TQ exerts these beneficial effects by efficiently increasing insulin-producing β-cells, up-regulating surviving VEGF, CD31, IL-10, GSH, and SOD, while down-regulating caspase-3, IL-1β, and TBARS in pancreatic tissues. The novel ability to suppress β-cell apoptosis and enhance islet revascularization in STZ-diabetic rats is particularly noteworthy (1-3).

Vitamin D deficiency has been closely associated with the onset of diabetes. Diabetes typically begins with the development of insulin resistance; cells may initially compensate for this resistance by secreting more insulin, which can prevent hyperglycemia. However, as this compensatory hyperactivity escalates, β-cells are negatively impacted by excess Ca^2+^ and reactive oxygen species (ROS), leading to cell death and the eventual onset of diabetes (4, 5).

Insulin resistance leads to a range of metabolic and cellular abnormalities, along with adverse clinical outcomes, many of which are classified under the diagnostic category of metabolic syndrome. Abnormalities associated with insulin resistance and compensatory hyperinsulinemia include hypertension, elevated plasma triglyceride levels, and reduced high-density lipoprotein (HDL) levels. Insulin resistance also increases the risk of type 2 diabetes, coronary heart disease, and other vascular complications such as retinopathy, nephropathy, and neuropathy. It is now understood that endothelial dysfunction is one of the earliest steps in the development of vascular complications linked to insulin resistance and metabolic syndrome (6, 7).

Vitamin D has been shown to stimulate insulin synthesis and secretion both *in vitro* and *in vivo*. Numerous studies on diabetes have reported that TQ significantly reduces blood serum glucose levels and that an inverse relationship exists between vitamin D levels and diabetes. Vitamin D3 and its analogs have been found to be effective against factors that play a major role in the pathogenesis of diabetes (8, 9). This study was planned to investigate the effect of thymoquinone (TQ) on vitamin D metabolism.

## Materials and Methods

### Animal materials

The study utilized 42 male Wistar albino rats weighing 150–200 g, obtained from the Van Yuzuncu Yil University, Experimental Application, and Research Center (Ethics Committee Approval No: 29/07/2021-2021/07-15).

### Preparation of experimental groups

The animals were randomly divided into six experimental groups, each consisting of seven rats: control (C), insulin resistance induced (I), thymoquinone administered (TQ), thymoquinone administered with insulin resistance induced (TQI), insulin resistance induced with thymoquinone administered (ITQ), insulin resistance induced with metformin for control treatment (IM). The rats were housed in cages with less than 12 hr of light/dark cycle, at a temperature of 22 ± 2 °C, and with continuous access to feed and fresh water throughout the 15-day experiment. 

An intraperitoneal injection of dexamethasone at a dosage of 1 mg/kg/day was administered to the rats for seven days to induce insulin resistance (10-13).

The normal standard feed consisted of the following composition: moisture (%12.8), crude protein (%23), crude fat (%2.7), crude fiber (%6.5), crude ash (%7.8), sodium (%0.4), dicalcium phosphate, calcium carbonate, sodium chloride, sodium bicarbonate, vitamin A, and a mixture of six microelements.

### Group assignments

Group 1 (Control-C): Seven randomly selected rats were assigned to the control group. The initial blood glucose level for this group was measured using blood collected from the tail. Physiological saline was administered intraperitoneally (IP) for 7 days, and sunflower oil was given by gavage during the last seven days.

Group 2 (Insulin Resistance-I): Seven rats were administered dexamethasone intraperitoneally (IP) for seven days, at a dosage of 1 mg/kg/day.

Group 3 (Thymoquinone-TQ): Thymoquinone at a dosage of 10 mg/kg/day was administered orally by gavage to seven rats for seven days.

Group 4 (Thymoquinone + Insulin Resistance-TQI): Thymoquinone at a dosage of 10 mg/kg/day was administered orally by gavage to seven rats for seven days. Following this, dexamethasone at a dosage of 1 mg/kg/day was administered via intraperitoneal injection for an additional seven days.

Group 5 (Insulin Resistance + Thymoquinone-ITQ): Dexamethasone at a dosage of 1 mg/kg/day was administered intraperitoneally (IP) to a group of seven rats for seven days. Thymoquinone at a dosage of 10 mg/kg/day was then administered orally by gavage for an additional seven days.

Group 6 (Insulin Resistance + Metformin-IM): Dexamethasone at a dosage of 1 mg/kg/day was administered intraperitoneally (IP) to a group of five rats for seven days. Metformin in powder form (40 mg/kg/day) was dissolved in water and administered orally by gavage for six days, with the trial concluding on the 10th day.

In all groups, glucose levels were measured using blood samples collected from the tail vein two hours after the final treatment (Biosensor glucose meter and strips, Lifechek, Turkey).

### Collection of blood samples

After the experimental period, blood was collected from the left ventricles of the hearts of the animals under ketamine anesthesia into gel glass serum tubes. The blood samples were then centrifuged at 3000 rpm at +4 °C for 15 min, and the separated serum samples were transferred into tubes.

### Tissue collection

In a sterile environment, approximately 200 mg of liver tissue was obtained from sacrificed rats using a sterile scalpel and forceps. The tissue was placed in 2 ml sterile RNase-free tubes, and an RNA stabilizer was added to completely cover it. The tubes containing the tissue were stored at -80 **°**C until total mRNA isolation.

### Preparation of tissue homogenate

The tissues stored in a deep freezer (-80 °C) were thawed at room temperature, and approximately 80 mg of the tissues were transferred to sterile tubes. To homogenize the tissues, 0.2 ml sterile phosphate buffer was added. The homogenized tissues were centrifuged at 1500 rpm at 4 °C. The liquid portion remaining at the top of the tube was discarded, and total RNA isolation was started immediately.

### Biochemical analyses

Blood glucose was determined in serum samples using a commercial kit on an auto-analyzer (FUJI DRI-CHEM NX500, Japan). 

For vitamin D analysis, 200 µl serum was transferred to plastic tubes and extracted. The device was calibrated using Vitamin D3 standards. Subsequently, 20 µl of the prepared extracts were analyzed by liquid chromatography (Agilent 1100 series HPLC) (14-16).

For serum insulin level determination, a Rat INS ELISA Kit (USBN Code Number: CEA448Ra, 96 Tests) was used, and readings were performed according to the kit’s protocol.

### Calculation of insulin resistance

HOMA-IR (Homeostasis Model Assessment of Insulin Resistance) is a widely used measure of insulin resistance, a condition in which the body’s cells respond less effectively to the hormone insulin. HOMA-IR is calculated based on the measurement of blood glucose and insulin levels. Elevated HOMA-IR values may indicate increased insulin resistance, which can raise the risk of developing type 2 diabetes and other metabolic disorders. Insulin resistance was calculated using the following formula (17,18).

HOMA IR= Plasma Glucose (mg/dl) × Fasting Plasma Insulin (mu/L)/405

### Gene expression analysis

The genes and characteristics examined in this study are summarized in Table 1.

### Total mRNA isolation and analysis

Real-time PCR was used to determine the expression of key genes involved in vitamin D metabolism from RNA isolation products obtained from rat liver tissue across all groups. Total RNA isolation was performed manually according to the protocol provided by geneAll (South Korea). cDNA was synthesized from the isolated RNAs using reverse transcriptase enzyme in a thermal cycle.

### Complementary DNA (cDNA) synthesis

According to the WizScriptTM cDNA Synthesis Kit protocol, the cDNA mixture was prepared in a 200 µl PCR tube. The cDNA mixture added to the tubes was distributed into PCR tubes. Reverse transcription was performed with the Rotor-Gene Q Software-Run device (with Wizscript cDNA synthesis program) according to the following program.

### RT-PCR analysis

The cDNAs obtained were diluted according to the dilution amount of each primer given in the stock for amplification with the help of primers designed specifically for the target gene region.

The cDNAs were amplified using a Qiagen-branded Rotor Gen real-time PCR device. According to the WizpureTMqPCR Master (SYBR) protocol, the following mixture was prepared for each sample using a 36-PCR tray. At the end of the PCR process, a Melting Curve, Ramp: 50-99 (1 degree increase), 90 ºC 5’’, was performed**.**

### Western blot analysis

Western blot assay was performed using the standard route. Protein quantification was performed using the Qubit® Protein Assay Kits (Thermo Fisher Scientific, Cat No: Q33211) kit using a Qubit® 3.0 Fluorometer (Thermo Fisher Scientific, Cat No: Q33216) according to the manufacturer’s instructions. Samples were loaded onto the gel, and SDS-page electrophoresis was performed. The iBLOT GEL TRANSFER System was used for blotting, and the nitrocellulose membrane was blocked in a blocking solution (5% BSA-PBST). A primary antibody was applied, and the membrane was washed with PBST. The secondary antibody was applied and washed with PBST. Membranes were taken in (NZY Supreme ECL HRP Substrate, Nzytech, Cat No: Mb19301) and placed in the imaging device (ChemIDoc-It2, UVP) and images were taken.

### Statistical analysis

The biochemical data of the groups obtained at the end of the study were evaluated by multiple comparison tests using the SPSS 22.0 software program. The raw values obtained from all analyses were presented as the mean ± standard error of the groups**.**

Glyceraldehyde 3-phosphate dehydrogenase (GAPDH) was used as a control gene to measure the expression levels of the genes. Each sample was analyzed in triplicate using qRT-PCR. Delta-Ct and delta-delta Ct values were used to analyze gene expression levels. A Ct (cycle threshold) was determined at the beginning of the logarithmic phase of amplifications. The difference in Ct values between the control group and repeated groups was used to determine the appropriate expression.

## Results

### Biochemistry results

Table 2 summarizes all biochemistry data measured in the blood samples collected after the experiment, including the substances assessed in the study groups.

The difference between the group means with different letters in the same row is considered significant (*P*<0.05).

Vitamin D concentration was found to be the lowest in the IMT group and the highest in the ID group. It was significantly lower in the TQ groups compared to the ID group (*P*<0.05). Insulin levels did not show a significant change in any group compared to the control group (*P*>0.05) but were lowest in the TQ group. No statistically significant difference was observed between the other groups. Blood glucose levels increased significantly in the ID group, which were higher than in all other groups (*P*<0.05). These levels decreased in the ITQ group compared to the ID group. HOMA-IR levels were the highest in the ID group (*P*<0.05), while no significant difference was found between the other groups (*P*>0.05). In the groups administered TQ, HOMA-IR levels were similar to those of the control group.

### RT-PCR results

Using RT-PCR, the expression levels of genes involved in vitamin D metabolism were determined and assessed. The results are shown in Table 3. According to RT-PCR data, the VDR gene expression increased 1.5-fold in the group given TQ for protection, 9-fold in the group given TQ for treatment, and 1.5-fold in the ID group. It was similar to the control in the TQ group and significantly down-regulated in the IMT group. No significant change was observed in the TQ group, VDBP was up-regulated 2-fold only in the IMT group and down-regulated in the ID, TQI, and ITQ groups. 

CALB1 was up-regulated 20-fold in the ID group and 8-fold in the TQ group, with IMT and TQI groups showing a lesser degree of up-regulation. ECaC1 was up-regulated 39-fold in the ITQ group, 4-fold in the ID group, and 2-fold in the IMT group, while it was significantly down-regulated in the TQ group and halved in the TQI groups. The CaBD-D9K was up-regulated 5-fold in the ID group, with a lesser up-regulation in the TQ groups. 

The Cyp27a1 gene was up-regulated 2-fold in the TQ group, 3-fold in the TQI group, 4-fold in the IMT group, and 6-fold in the ITQ group. The Cyp2R1 gene showed no significant change in the ID and IMT groups but was down-regulated by half in the ITQ and TQI groups, with the greatest down-regulation in the TQ group. The Cyp27b1 gene increased 4-fold in the TQ group and 1.5-fold in the TQI group, while it was down-regulated by half in the IMT and ID groups and remained similar to the control in the ITQ group. The Cyp24a1 gene increased 2-fold in the TQI and IMT groups, 2.5-fold in the ID group, 2-fold in the IMT group, and 1.5-fold in the TQ group, and was minimally down-regulated in the ITQ group.

### Western blot (WB) analysis results for the study groups

Following RT-PCR analyses of the genes involved in vitamin D metabolism, WB analyses were also conducted for the same genes, which were also performed. The results from these analyses are presented below (Table 4).

**Figure 1:**
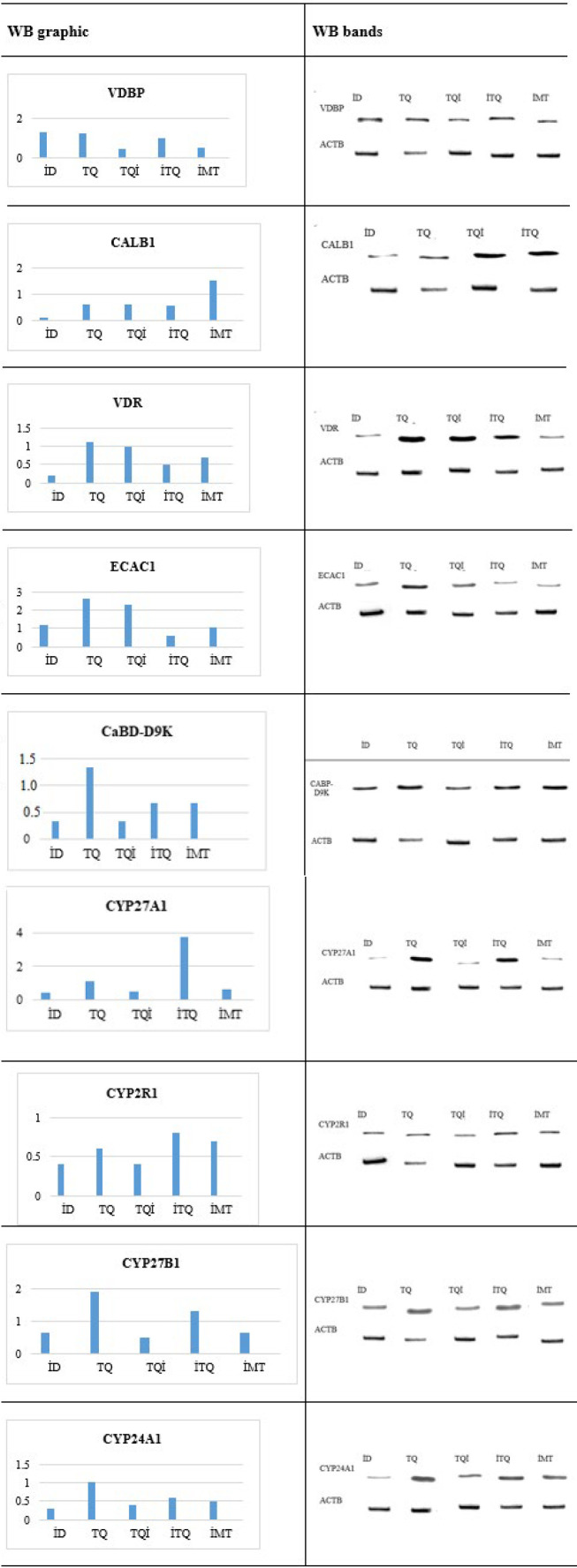
Graphic and band images of western blot results on the effect of thymoquinone on vitamin D metabolism genes in insulin-resistant rats

Figure 1 shows the protein transformation of genes involved in vitamin D metabolism as a result of WB analyses. WB graphs were created according to the control group. Therefore, the results of the five groups are presented.

Western blot analysis revealed the conversion of gene expression into protein related to vitamin D metabolism. VDBP levels slightly increased in the TQ and ID groups remained similar to the control in the ITQ group and were reduced by half in the other groups. ECaC1 protein expression showed a notable increase in the TQ and TQI groups, while it decreased in the ITQ group. CALB1 levels were up-regulated 1.5-fold in the IMT group but exhibited the greatest decrease in the ID group. VDR protein expression remained comparable to control levels in the TQ and TQI groups but significantly decreased in the other groups, with the largest reduction observed in the ID group. CaBP-D9K levels increased slightly in the TQ group but declined in the other groups. Cyp27a1 protein levels remained unchanged in the TQ group, increased 3.8-fold in the ITQ group, and decreased in the ID, TQI, and IMT groups. Cyp27b1 levels increased by 1.9-fold in the TQ group and 1.3-fold in the ITQ group, while they were halved in the other groups. Lastly, Cyp24a1 expression remained stable in the TQ group but decreased most prominently in the ID group and by half in the other treatment groups. These findings demonstrate the variable impact of TQ and other treatments on protein expression related to vitamin D metabolism, emphasizing potential therapeutic implications in the management of insulin resistance.

The comparison between RNA transcription levels of genes involved in vitamin D metabolism and their corresponding protein expression levels, as determined by WB analyses, revealed the following results: VDBP showed the highest gene expression in the IMT group. However, its protein conversion rate differed significantly across the groups. CALB1 had the highest gene expression in the ID group, with consistent protein conversion across all groups. VDR exhibited the highest gene expression in the ITQ group, but its protein conversion rate differed markedly compared to other groups. ECaC1 expression was highest in the ITQ group, with notable differences in protein conversion between the TQ and ITQ groups. CaBP-D9K gene expression peaked in the ID group, while its protein conversion rate in the TQ group was significantly different from the others. Cyp27a1 gene expression was highest in the ITQ group, like proteine conversion. Cyp2R1 had the highest gene expression in the ID and IMT groups, but protein conversion rates varied significantly in the TQI, ID, and IMT groups. Cyp27b1 expression peaked in the TQ group, with notable differences in protein conversion between the TQI and IMT groups compared to the ID and ITQ groups. Cyp24a1 exhibited the highest gene expression in the ID group, with considerable variation in protein conversion in the TQI, ID, and IMT groups.

These findings underscore the discrepancies between RNA transcription levels and protein conversion rates among different treatment groups, suggesting complex regulatory mechanisms in vitamin D metabolism that TQ and other treatments influence.

## Discussion

Several studies have explored the antidiabetic mechanisms of thymoquinone (TQ). In one investigation using an experimental type 2 diabetes mellitus (T2DM) model, TQ treatment was found to reduce hyperglycemia-induced insulin resistance significantly. The study demonstrated that TQ markedly lowered elevated glucose levels, glucose AUC, insulin levels, HOMA-IR, and DPP-IV levels. These findings suggest that TQ mitigates hyperglycemia-associated insulin resistance by enhancing insulin sensitivity, improving lipid profiles, and inhibiting DPP-IV activity (3). 

A study investigating the effects of TQ on intracellular signaling pathways revealed that proliferative concentrations of TQ utilized the PI3K/AKT/mTOR pathway to regulate NRK-52E cell proliferation. In the same study, higher TQ concentrations were found to inhibit cell proliferation through increased receptor tyrosine kinase (RTK) signaling (19). Additionally, studies have reported that TQ and lycopene significantly contribute to maintaining oxidative balance in the BHK cell line model (20). TQ reduces gene expression values affected by anti-oxidant effects and exposure to ionizing radiation (21).

In this study, while insulin levels were not significantly different from the control in all experimental groups, blood glucose levels were notably higher in the insulin resistance (ID) group compared to all other groups. Interestingly, the TQ treatment group (ITQ) exhibited lower blood glucose levels than the control group (Table 2). HOMA-IR levels, a key indicator of insulin resistance, were significantly elevated in the ID group but decreased in the TQ-treated groups, approaching control values (Table 2). These results suggest that TQ may have a beneficial effect on improving insulin resistance and promoting glucose homeostasis.

Serum vitamin D levels were significantly elevated in the ID group (*P*≤0.05), aligning with the known effects of dexamethasone, which potentiates calcitriol actions by inhibiting cell growth and enhancing vitamin D receptor (VDR) expression and VDR-mediated transcription (22). However, in the groups receiving TQ for prevention and treatment, vitamin D levels decreased and approached control levels, suggesting that TQ may play a role in normalizing vitamin D metabolism. Gene expression analysis showed a slight up-regulation (1.5-fold) of VDR, the gene coding for the vitamin D3 receptor protein, in the insulin-resistant group. However, TQ treatment markedly up-regulated VDR expression nine times in the treatment group (ITQ). This divergence from previous findings (22) suggests that TQ may exert a unique regulatory effect on VDR expression, potentially influencing vitamin D-mediated pathways in a distinct manner.

The down-regulation of VDBP, which binds and transports vitamin D and its metabolites, in all insulin-resistance groups (ID, TQI, and ITQ) suggests impaired vitamin D metabolism in liver tissue. CALB1, a calcium-binding protein, showed a 20-fold increase in the insulin-resistant group yet normalized in the TQ-treated groups, aligning with serum vitamin D levels. Interestingly, despite the high expression of CALB1, CaBP-D9K, and ECAC1 in insulin resistance, their lower protein conversion rates suggest that these proteins may function independently of vitamin D-related mechanisms.

In this study, treatment with TQ was shown to significantly influence the expression of genes associated with vitamin D metabolism. Notably, CYP27A1, which catalyzes the 25-hydroxylation of vitamin D3, exhibited up-regulation in TQ-treated groups. This finding suggests that the observed effects may stem from TQ metabolism rather than direct involvement in the modulation of insulin resistance. Conversely, CYP2R1, a key microsomal vitamin D hydroxylase, remained unchanged in response to insulin resistance. Additionally, while CYP27B1, responsible for synthesizing the active form of vitamin D3, was down-regulated in the insulin resistance group, its expression normalized in TQ-treated groups. It was negatively correlated with serum vitamin D levels. Furthermore, CYP24A1, which plays a crucial role in the regulation of active vitamin D, demonstrated increased expression in the context of insulin resistance.

Previous studies have indicated that vitamin D receptors may play a critical role in modulating glucose parameters, lipid metabolism, and other protective effects in diabetic models (23). Notably, low serum vitamin D levels were significantly associated with elevated HOMA-IR levels in individuals with type 2 diabetes. Vitamin D deficiency has been shown to exacerbate inflammation via the NF-kB pathway, thereby increasing insulin resistance (24). Additionally, the indirect anti-oxidant properties of vitamin D contribute to the regulation of reactive oxygen species (ROS), further underscoring its potential role in the management of insulin resistance (25).

**Table 1:** Genes involved in vitamin D metabolism, their symbols, and primary sequences

Gene name	Gene symbol	Primer name F (5’-3’)	Primer name R (5’-3’)
Vitamin D binding protein	VDBP	GTGCTGCTCCATAAACTCTCCT	GCCATCTCTGTGGTATTTGCTTG
Calbindin 1	Calb1	CAGGATGGGCAACGGATACATAGA	TGTCCCCAGCAGAGAGAATAAG
Vitamin D receptor	VDR	TGCCGCCTGTCTGTGTTATTCT	GGTTCGTGTGGTAGTGTATTTTGG
Transient Receptor Potential Cation Channel, Subfamily V, Member 5	ECaC1	ACCTATCTCTGCTTCTTCTGACC	ACTGTTGGAGGGAGTTGGATTT
S100 calcium binding protein G	CaBP-D9K	TACAGCGAGTGCCTCAAGTT	ATGTGGATGTAGTTGCGCGT
Cytochrome P450 Family 27 Subfamily A Member 1	Cyp27a1	CCAGTTATACCGCTCTGGTT	ACTGCTGTCCTTGTGTGATGA
Cytochrome P450 family 2 subfamily R member 1	Cyp2R1	GGCTCGGATGGAAATGTTCT	TGTGCTCTTTCAGCGTCTTT
Cytochrome P450 Family 27 Subfamily B Member 1	Cyp27b1	ACTTCGCACAGTTTATGTGGC	ATTCTTCACCATCCGCCGTT
Cytochrome P450 Family 24 Subfamily A Member 1	Cyp24a1	ATGGGACAACCTGCGAAACA	ACGTCACAAAGATGGAGCGA

**Table 2:** Biochemical parameters on the effect of thymoquinone on vitamin D metabolism genes in insulin-resistant rats

Groups	Vitamin D (µg/ml)	Insulin (pq/ml)	Glucose(mg/dl)	HOMA-IR
C group	0.0420±0.0039ab	1076±174.74a	214.5±16.6bc	544±61.4b
ID group	0.0517±0.0058a	1318±306.5ab	336±58.35a	1080±293.8a
TQ group	0.025±0.004b	799±283.055a	231±20.3b	420±122.6b
TQI group	0.0280±0.005b	1396±158.7ab	186±18.4bc	682±125.6ab
ITQ	0.0282±0.008b	1434±298.9ab	127±9.2c	443±90.75b
IMT	0.0095±0.001bc	2329±372.5ab	143±33.3bc	787±120.09ab

**Table 3:** Gene expression results on the effect of thymoquinone on vitamin D metabolism genes in insulin-resistant rats

	VDBP	CALB1	VDR	ECAC1	CABP-D9K	CYP27A1	CYP2R1	CYP27B1	CYP24A1
Control	1	1	1	1	1	1	1	1	1
ID	0.37	20	1.5	4	5	1.4	1.3	0.5	2.5
TQ	0.94	7.8	0.8	0.06	0.4	2	0.06	4	1.5
TQI	0.26	1.6	1.5	0.6	3.5	3	0.8	1.5	2
ITQ	0.44	3.8	9	39	2	6	0.5	1	0.4
IMT	2	1.5	0.05	2	2	4	1.2	0.5	2

**Table 4:** Western blot results on the effect of thymoquinone on vitamin D metabolism genes in insulin-resistant rats

	VDBP	CALB1	VDR	ECAC1	CABP-D9K	CYP27A1	CYP2R1	CYP27B1	CYP24A1
Control	1	1	1	1	1	1	1	1	1
ID	1.3	0.14	0.2	1.2	0.4	0.4	0.4	0.65	0.3
TQ	1.2	0.63	1.1	2.6	1.31	1.08	0.6	1.9	1
TQI	0.4	0.61	0.98	2.3	0.4	0.5	0.4	0.5	0.4
ITQ	0.95	0.58	0.5	0.6	0.7	3.8	0.8	1.3	0.6
IMT	0.5	1.5	0.7	1.04	0.7	0.6	0.7	0.65	0.5

WB: Western blot

**Figure 2:**
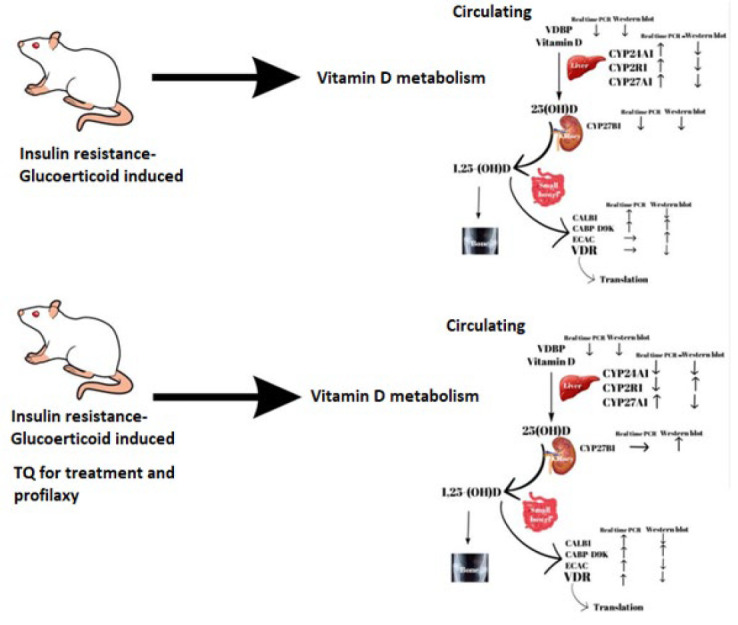
Graphical abstract for results on the effect of thymoquinone on vitamin D metabolism genes in insulin-resistant rats

## Conclusion

This study demonstrates that TQ exerts beneficial effects against insulin resistance, serving both as a preventive and therapeutic agent, thereby highlighting its antidiabetic properties. While serum vitamin D levels were elevated in the experimental insulin resistance group, TQ treatment effectively normalized these levels, suggesting a significant interaction between TQ and vitamin D metabolism. Gene expression analysis indicates that TQ influences vitamin D metabolism within liver tissue, particularly through modulation of vitamin D receptor (VDR) and associated genes. However, the limited impact on certain genes suggests a complex interplay, with these genes likely playing roles in vitamin D metabolism in other tissues (Figure 2).

Future research is warranted to elucidate the detailed mechanisms of action in various tissues and to gather more comprehensive data. Overall, TQ presents considerable promise as a therapeutic agent for enhancing metabolic health and managing insulin resistance.

These findings imply that TQ’s therapeutic effects in managing insulin resistance may be intricately linked to its modulation of vitamin D metabolism. Future research is warranted to elucidate the detailed mechanisms of action in various tissues and gather more comprehensive data. Overall, TQ presents considerable promise as a therapeutic agent for enhancing metabolic health and managing insulin resistance.

## Data Availability

The data that support the findings of this study are available from the corresponding author, upon reasonable request.

## Declaration

We have not used any AI tools or technologies to prepare this manuscript.
